# Sulforaphane and TRAIL induce a synergistic elimination of advanced prostate cancer stem-like cells

**DOI:** 10.3892/ijo.2014.2335

**Published:** 2014-03-10

**Authors:** SABRINA LABSCH, LI LIU, NATHALIE BAUER, YIYAO ZHANG, EWA ALEKSANDROWICZ, JURY GLADKICH, FRANK SCHÖNSIEGEL, INGRID HERR

**Affiliations:** Experimental Surgery, General, Visceral and Transplantation Surgery, University of Heidelberg, D-69120 Heidelberg, Germany

**Keywords:** cancer stem cells, prostate cancer, apoptosis resistance

## Abstract

Advanced androgen-independent prostate cancer (AIPC) is an aggressive malignancy with a poor prognosis. Apoptosis-resistant cancer stem cells (CSCs) have been identified in AIPC and are not eliminated by current therapeutics. Novel therapeutic options, which are currently being evaluated in patient studies, include TRAIL and the broccoli-derived isothiocyanate sulforaphane. Although neither agent targets normal cells, TRAIL induces apoptosis in most cancer cells, and sulforaphane eliminates CSCs. In this study, the established AIPC cell lines DU145 and PC3, with enriched CSC features, and primary patient-derived prostate CSCs were treated with sulforaphane and recombinant soluble TRAIL. We examined the effects of these drugs on NF-κB activity, self-renewal and differentiation potential, and stem cell signaling via spheroid- and colony-forming assays, FACS and western blot analyses, immunohistochemistry, and an antibody protein array *in vitro* and after xenotransplantation. We largely found a stronger effect of sulforaphane on CSC properties compared to TRAIL, though the agents acted synergistically when applied in combination. This was associated with the inhibition of TRAIL-induced NF-κB binding; CXCR4, Jagged1, Notch 1, SOX 2, and Nanog expression; ALDH1 activity inhibition; and the elimination of differentiation and self-renewal potential. *In vivo*, tumor engraftment and tumor growth were strongly inhibited, without the induction of liver necrosis or other obvious side effects. These findings suggest that sulforaphane shifts the balance from TRAIL-induced survival signals to apoptosis and thus explains the observed synergistic effect. A nutritional strategy for high sulforaphane intake may target the cancer-specific activity of TRAIL in CSCs.

## Introduction

Prostate cancer is the most commonly diagnosed cancer and second leading cause of cancer-related death in men in Western countries (1). The most common treatment is hormone deprivation; however, during disease progression, the malignant cells lose their androgen receptors and thereby their hormone dependence and therapeutic responsiveness. The resulting androgen-independent prostate cancer (AIPC) progresses and metastasizes, with no effective therapeutic options at present (2). Although the cellular composition of prostate cancer is heterogeneous, AIPC cells are suggested to possess cancer stem cell (CSC) characteristics (3). According to the hypothesis, CSCs mediate tumor formation, progression, and metastasis and do not respond to chemoor radiotherapy (4); therefore, CSCs may be enriched by treatment, increasing resistance in late-stage cancer. Due to their ability for self-renewal and differentiation, CSCs can generate all the cellular subtypes of the original tumor. In addition, these cells overexpress CSC markers, including ALDH1, CD44, CD133 and c-Met (3).

Human tumor necrosis factor (TNF)-related apoptosis ligand (TRAIL) has achieved promising therapeutic results by targeting only malignant cells and leaving normal cells undamaged. Although phase 1/2 clinical trials have demonstrated a favorable toxicity profile for recombinant soluble TRAIL, there is limited evidence of antitumor activity (5); this may be due to the short exposure of tumor cells to low concentrations of TRAIL, which has a short half-life. Moreover, TRAIL signaling does not always cause apoptosis in cancer cells. Indeed, some studies have shown that TRAIL may induce a prosurvival response via signaling factors that include nuclear factor (NF)-κB, mitogen-activated protein kinase (MAPK), and Akt (5). This finding is corroborated by data obtained in prostate cancer and glioblastoma that have indicated that some cancer cells are resistant to TRAIL; it has been demonstrated that these cells are CSCs (6). A recent study proposes that the dietary agent sulforaphane, a mustard oil and isothiocyanate present in high concentrations in broccoli and cauliflower (7), sensitizes prostate cancer cells to TRAIL-induced apoptosis (8), though the authors did not focus on CSCs. Our recent results demonstrate that sulforaphane sensitizes resistant pancreatic CSCs to TRAIL-induced apoptosis through the inhibition of basal and TRAIL-induced NF-κB activity (9). Based on several promising animal and epidemiological studies, prospective clinical trials with sulforaphane-enriched broccoli sprout extracts are ongoing in the US to examine the effects on atypical nevi and bladder and prostate cancer (10). A pilot study at our clinic has been initiated to evaluate the effects of sulforaphane-enriched broccoli sprouts on patients with advanced pancreatic cancer.

The present study utilized the DU145 and PC3 cell lines, with highly enriched CSC features, and primary prostate CSCs to demonstrate that colony- and spheroid formation is strongly affected by sulforaphane, whereas TRAIL had only minor activity in targeting CSC features. However, the combination of both agents acted synergistically, indicating that sulforaphane shifted the balance of TRAIL-induced survival and suicide signaling to the cell death pathway. The results may be due to the sulforaphane-mediated inhibition of NF-κB activity and stem cell signaling, including tumor engraftment and growth *in vivo*. Our data provide new mechanistic insight into the synergistic effects of TRAIL and sulforaphane on prostate CSCs.

## Materials and methods

### Tumor cell lines

PC3 and DU145 prostate cancer cell lines were obtained from American Type Culture Collection (Manassas, VA, USA) and authenticated throughout culture by the typical morphology. The cells were cultured in RPMI-1640 (PAA, Pasching, Austria) supplemented with 10% heat-inactivated FCS (Sigma, Deisenhoffen, Germany) and 25 mmol/l HEPES (PAA). Human prostate CSCs were obtained from CELPROGEN (San Pedro, CA, USA) and cultivated in ‘Human Prostate Cancer Complete Growth Medium’ (CELPROGEN) on ‘Human Prostate Cancer Stem Cell Matrix’-coated tissue containers (CELPROGEN). To maintain the authenticity of the cell lines, frozen stocks were prepared from the initial stocks, and a new frozen stock was thawed every three months for the experiments. The established cell lines were recently authenticated by a commercial service (Multiplexion, Heidelberg, Germany). Mycoplasma-negative cultures were ensured by monthly mycoplasma tests.

### Cytotoxic agents

D, L-Sulforaphane (Sigma-Aldrich, St. Louis, MO, USA) was dissolved in ethanol to generate a 100 mM stock. Recombinant Super Killer TRAIL was obtained from AXXORA (Lörrach, Germany) and was dissolved in TRAIL buffer AXXORA (Lörrach) to generate a 100 *μ*g/ml stock solution. The final concentrations of solvents in the media were ≤0.1%.

### Gel retardation analysis of NF-κB binding

The preparation of nuclear protein extracts and the bandshift reaction using the Light Shift^®^ Chemiluminescent EMSA kit were performed as we recently described (11).

### Colony-forming assay

Treated cells were seeded at a cell density of 500 cells/well in complete medium in 6-well tissue culture plates (TPP), and the colony-forming assay was performed as we recently described (12).

### Spheroid assay

For spheroid formation, the cells were cultured in NeuroCult NS-A basal serum-free medium (human) (StemCell Technologies, Vancouver, Canada) supplemented with 2 *μ*g/ml Heparin (StemCell Technologies), 20 ng/ml hEGF (R&D Systems, Wiesbaden-Nordenstadt, Germany), 10 ng/ml hFGF-b (PeproTech, Hamburg, Germany), and NeuroCult NS-A Proliferation Supplements (StemCell Technologies). For the evaluation of the first generation of sphere formation, the cells were seeded at clonal density (5×10^2^ cells/ml) in 12-well low-adhesion plates in 1 ml medium per well. Upon sphere formation, the spheres were dissociated, and the cell number was evaluated. The viable cells were reseeded under the same conditions as mentioned above to evaluate the potential of secondary sphere formation.

### ALDH1 activity

ALDEFLUOR substrate (5 *μ*l; Aldagen, Inc., Durham, NC, USA) was added to 1×10^6^ treated PC3 cells in 500 *μ*l assay buffer and incubated for 60 min at 37°C. Pre-treatment with the ALDH1 inhibitor diethylaminobenzaldehyde was used as a negative control.

### Western blot analysis

Following treatments, the proteins were isolated, and a western blot analysis was performed as described (9). The following antibodies were used: mouse mAbs against human Notch 1, CXCR4 (Abcam, Cambridge, UK), and β-actin (Sigma-Aldrich) and rabbit polyclonal Abs against human Jagged1 (Abcam), SOX2 and Nanog (Cell Signaling, Danvers, MA, USA).

### Human pluripotent stem cell antibody array

Nitrocellulose membranes on which capture antibodies had been spotted and the detection reagents were obtained as a kit from R&D Systems^®^ (R&D Systems). According to the instructions of the manufacturer, the protein extracts were harvested after cell lysis and incubated overnight with the nitrocellulose membranes. After washing, the membranes were incubated with biotinylated secondary antibodies and streptavidin-HRP and chemiluminescent detection reagents were used to detect binding.

### Adipogenic differentiation assay

Tumor cells (1×10^5^) were seeded and treated in 6-well plates. To induce adipogenic differentiation, the medium was changed to NH AdipoDiff Medium, 2 ml per well. The medium was refreshed every third day; after 14 days, the fat droplets of adipogenic cells were stained with Oil Red O (Sigma-Aldrich).

### Transplantation of tumor cells on fertilized chicken eggs

This assay was performed as described recently (13), but with modifications. Fertilized white leghorn chicken eggs (Geflügelzucht Hockenberger, Eppingen, Germany) were incubated at a humidity of 45–55% and 37.8°C in digital motor breeders Type 168/D incubator (Siepmann GmbH, Herdecke, Germany). At day 4 of embryonic development, 2–3 ml of albumen was removed with a syringe, allowing detachment of the embryo. A small window was cut into the eggshell and then sealed with tape. At day 8 of embryonic development, small handmade rings from Thermanox™ cover discs (Thermo Scientific, Schwerte, Germany) were placed on the CAM, and 1×10^6^ pre-treated or untreated tumor cells mixed with Matrigel at a ratio of 1:1 were deposited into the rings of the viable embryos. For the *in ovo* treatment of xenografts, a Whatman paper saturated with sulforaphane solution (10 *μ*M) was deposited next to untreated tumors at day 11. At day 12, a TRAIL solution (5 ng/ml) was dropped onto the Whatman paper until it was saturated. At day 18, the xenografts were resected to determine the tumor engraftment rate and the tumor volume. All the embryos that died before day 18 were excluded from further analyses. The tumor volumes were estimated by the following formula: Volume = 4/3 × ∏ × r^3^ (r = 1/2 × square root of diameter 1 × diameter 2) (13).

### Immunofluorescence staining of tumor xenograft tissue

For immunofluorescence staining, frozen xenograft tissue was sectioned, and the staining was performed according to a standard protocol. In short, the tissue was fixed in 4% PFA for 10 min; rabbit polyclonal Abs against human CD44 (Gene Tex, Irvine, CA, USA), CXCR4, and c-Met (Abcam) and mouse polyclonal Abs against RelA (Rockland, Gilbertsville, USA), Nanog (Cell Signaling), EpCam (kindly provided by Dr G. Moldenhauer), ALDH (Becton-Dickinson, Heidelberg, Germany), and CD133 (Millipore, Bergisch Gladbach, Germany) were used as the primary Abs. The nuclei were stained with DAPI (4,6-diamidino-2′-phenylindol, 1 *μ*g/ml). Goat anti-rabbit Alexa Fluor 488 IgG, goat anti-rabbit Alexa Fluor 594 IgG, goat anti-mouse Alexa Fluor 594 IgG, and goat anti-mouse Alexa Fluor 488 IgG (Invitrogen, Camarillo, CA, USA) were used as the secondary Abs.

### Immunohistochemical staining of tumor xenograft tissue

For the immunohistochemical staining of primary spheroidal cultures and xenograft tissue, the Avidin/Biotin blocking kit (Vector, Burlingame, CA, USA) was used according to the instructions of the manufacturer. Endogenous peroxidase was quenched by 0.3% H_2_O_2_ in methanol. The primary Abs used are described above in the immunofluorescence section. Biotinylated goat anti-rabbit or anti-mouse IgG (Vector) served as the secondary Abs. The signal was enhanced using the ABC Elite kit (Vector). The samples were counterstained with hematoxylin (Dako, Glostrup, Denmark) and mounted in Pro Tags Mount Aqua (Quartett, Berlin, Germany). Omission of the primary Abs served as a negative control. The signals for immunohistochemistry and immunofluorescence staining were detected at ×400 magnification using a Leica DMRB fluorescence microscope (Leica, Wetzlar, Germany). The images of representative fields were obtained using a SPOT™ FLEX 15.2 64-Mp pixel-shifting digital color camera (Diagnostic, Instruments, Inc. USA) and analyzed with SPOT Basic/Advanced 4.6 software.

### H&E histochemical staining of liver sections

Liver tissue obtained from the chicken embryos was sectioned and stained with eosin (Sigma-Aldrich) and hematoxylin (Dako) following a standard protocol. The signal was detected at ×400 magnification using a Leica DMRB microscope (Leica). The images of representative fields were obtained using a SPOT FLEX 15.2 64-Mp pixel-shifting digital color camera (Diagnostic, Instruments, Inc.) and analyzed with SPOT Basic/Advanced 4.6 software.

### Statistical analysis

The quantitative data are presented as the mean ± SD. The data were analyzed using Student’s t-test for statistical significance. Variances in the tumor volumes were evaluated with the Kruskal-Wallis test and Mann-Whitney test with the Bonferroni correction. ^*^p<0.05 was considered statistically significant and ^**^p<0.001 as statistically highly significant.

## Results

### The combination of sulforaphane and TRAIL is superior to single treatments in reducing self-renewal potential

**Figure 1.**
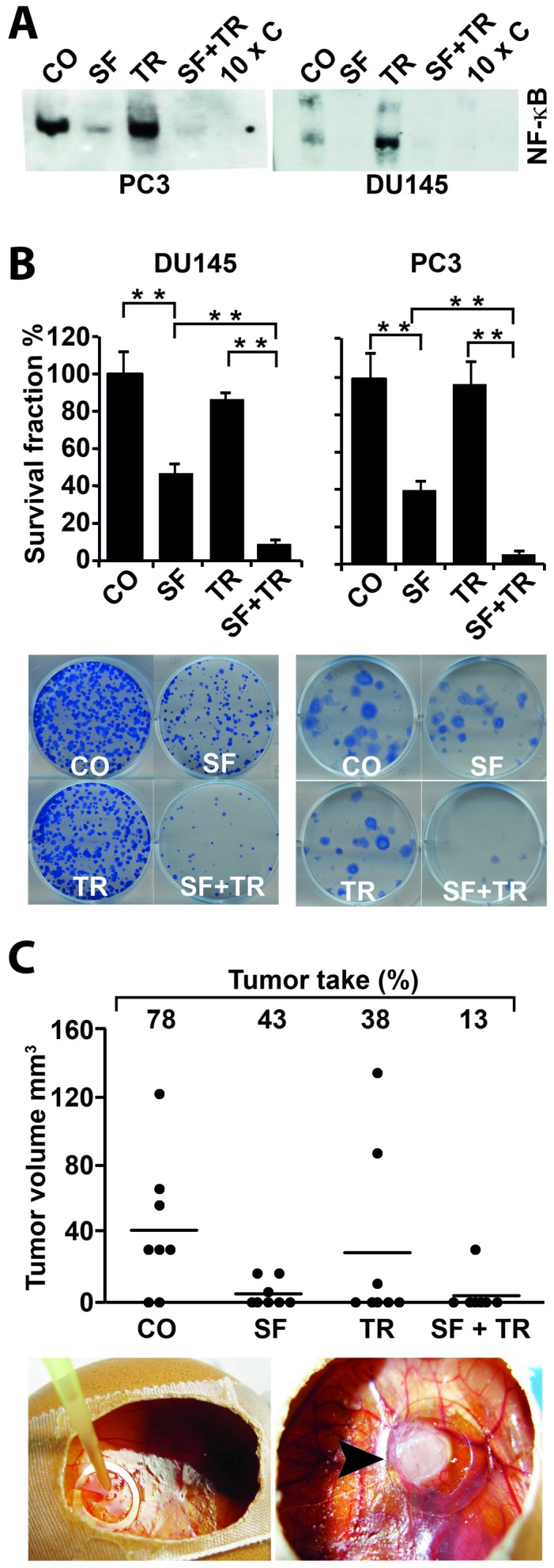
The combination of sulforaphane and TRAIL is superior to single treatments in reducing self-renewal potential. (A) DU145 and PC3 cells were left untreated or were treated with 10 *μ*M sulforaphane; after 24 h, TRAIL was added at a concentration of 5 ng/ml. After an additional 24 h, the nuclear proteins were harvested and DNA binding was analyzed by EMSA using a biotin-labeled oligonucleotide probe for the NF-κB promoter consensus sequence. The specific NF-κB shifts are marked. Competition with a 10-fold excess of unlabeled oligonucleotide (10 × C) served as a control for the binding specificity. (B) Twenty-four hours after TRAIL treatment, the cells were trypsinized and re-plated in a normal medium at a low density (500 cells/well) in 6-well plates. Ten days later, the cells were stained, and colonies containing >50 cells were counted under a dissecting Zeiss Stemi DV4 microscope. Images of the fixed and stained colonies are presented in the lower panel. The data are presented as the mean of three independent experiments, and SD are shown (^*^p<0.05, ^**^p<0.01). (C) Following *in vitro* treatment as described above, PC3 cells were transplanted onto the chorioallantoic membrane of fertilized chicken eggs at day 8 of embryonic development. Nine days later (day 17), the developed xenograft tumors were resected, and the tumor engraftment rates and tumor volumes were evaluated. The tumor volumes are presented as black dots and tumor engraftment is presented as the percentage of grown tumors relative to the number of each treatment group. Below the diagram, representative images show the transplantation of the tumor cells into a plastic ring on the CAM (left images), and a developed PC3 xenograft is marked with an arrow (right image).

**Figure 2.**
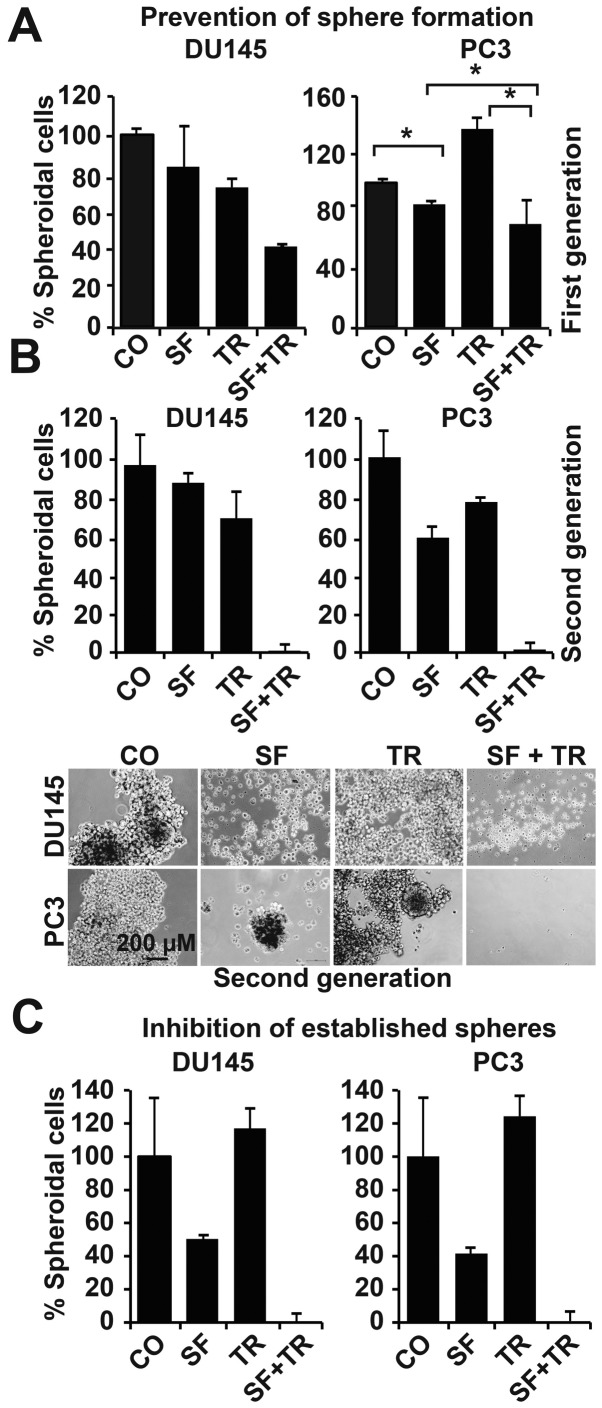
The combination of TRAIL and sulforaphane synergistically inhibits spheroid formation. (A) DU145 and PC3 cells were treated as described in Fig. 1A. At 24 h after TRAIL treatment, the cells were seeded at clonal density (5×10^2^ cells/ml) in 12-well low-adhesion plates in NSA-culture medium to support anchorage-independent growth. Seven days later, the formation of spheroids was determined by dissociating the spheroidal-growing cells and counting the number of viable cells, which is given as the percentage of spheroidal cells (first generation). The number of spheroidal cells in the control was set to 100%. (B) The surviving first-generation cells were re-seeded at clonal density in low-adhesion plates. Seven days later, when spheroid formation occurred, the spheroids were dissociated and the number of viable cells was counted (second generation). Representative images of second-generation spheres are shown in the lower panel. (C) Cells were seeded at clonal density in low-adhesion plates in NSA-medium; the full-grown spheres were treated as described in Fig. 1A after 3 days. Seven days later, the spheres were dissociated and the number of viable cells was evaluated. The data are presented as the mean of three independent experiments, and SD are shown (^*^p<0.05, ^**^p<0.01).

As model cell lines, we used the human prostate cell lines DU145 and PC3, which are derived from advanced AIPC metastases. These cells harbor highly enriched CSC characteristics (9,14–25), as summarized in Table I. We performed an electrophoretic mobility shift assay (EMSA) to measure the effect of TRAIL and sulforaphane on NF-κB activity. PC3 and DU145 cells were pre-treated with sulforaphane for 24 h to mimic a steady-state level of nutritional uptake, and the cells were then treated with TRAIL for an additional 24 h - either alone or in combination. This treatment schedule was used in all the following assays. Both cell lines exhibited basal NF-κB activity, which was higher in the PC3 cells and further increased by TRAIL (Fig. 1A). Sulforaphane inhibited this basal NF-κB activity and completely prevented TRAIL-induced NF-κB activity in the combination treatment. To examine the effect on self-renewal potential, the cells were seeded at a low density after treatment, and the formation of colonies was evaluated after 10 days (Fig. 1B). Sulforaphane significantly reduced clonogenic cell division to ∼50%, whereas TRAIL had only minor effects; however, synergism occurred in the combination treatment, as colony formation was almost completely inhibited. To confirm these results *in vivo*, we xenotransplanted untreated or *in vitro*-treated PC3 cells to the chorioallantois membrane (CAM) of fertilized chicken eggs; nine days later, the self-renewal capacity was evaluated by determining of the ability to form tumors *in vivo*, a feature attributed to CSCs (Fig. 1C). Sulforaphane reduced the rate of tumor engraftment of the untreated control cells from 78 to 43%, TRAIL to 38%, and the double treatment to 13%. The average volume of the tumors that developed in each group was 40, 7, 15 and 2 mm^3^ for the control, sulforaphane, TRAIL and combination groups, respectively. To further investigate these results, the self-renewal potential of the cells was evaluated by an analysis of the anchorage-independent spheroidal growth, another typical feature of stem cells. Two different types of sphere assays were performed to measure: i) the effect of treatment on spheroid formation, and ii) the effect of treatment on established spheroids. In the first assay, we seeded pretreated cells at clonal density under conditions that favor the proliferation of stem cells; seven days later, the number of spheroidal-growing cells was determined by dissociating the spheroids and counting the viable cells (Fig. 2A). The percentage of spheroidal-growing cells was reduced by sulforaphane in both cell lines to ∼80%; TRAIL also led to a reduction of 80% in the DU145 cells but to enhanced spheroid formation of 130% in the PC3 cells. The combined treatment significantly further reduced the percentage of spheroidal-growing cells to 40% in the DU145 cells, whereas the reduction was not significant in the PC3 cells. Furthermore, the cells were damaged by the combined treatment, which was demonstrated by re-seeding equal numbers of the cells at clonal density for spheroid formation without additional treatment and determining the number of spheroidal-growing cells after seven days (Fig. 2B). A substantial number of cells derived from the sulforaphaneor TRAIL-treated groups were still able to form spheroids, whereas none of the cells derived from the combination group formed spheroids. In a third test, we evaluated the efficacy of the treatment in reducing the percentage of cells derived from established spheres (Fig. 2C). The cells were seeded at clonal density for spheroid formation and treated three days later; the efficacy was evaluated after an additional seven days. Sulforaphane alone reduced the percentage of spheres to ∼40%, whereas TRAIL slightly induced sphere formation in both cell lines. Moreover, the co-treatment completely destroyed sphere formation. These results demonstrate that the combination of sulforaphane and TRAIL, but not the single agents, is sufficient to completely eliminate the self-renewal potential of DU145 and PC3 cells.

### The combination of sulforaphane and TRAIL is superior to single treatments in reducing CSC-related signaling

**Figure 3.**
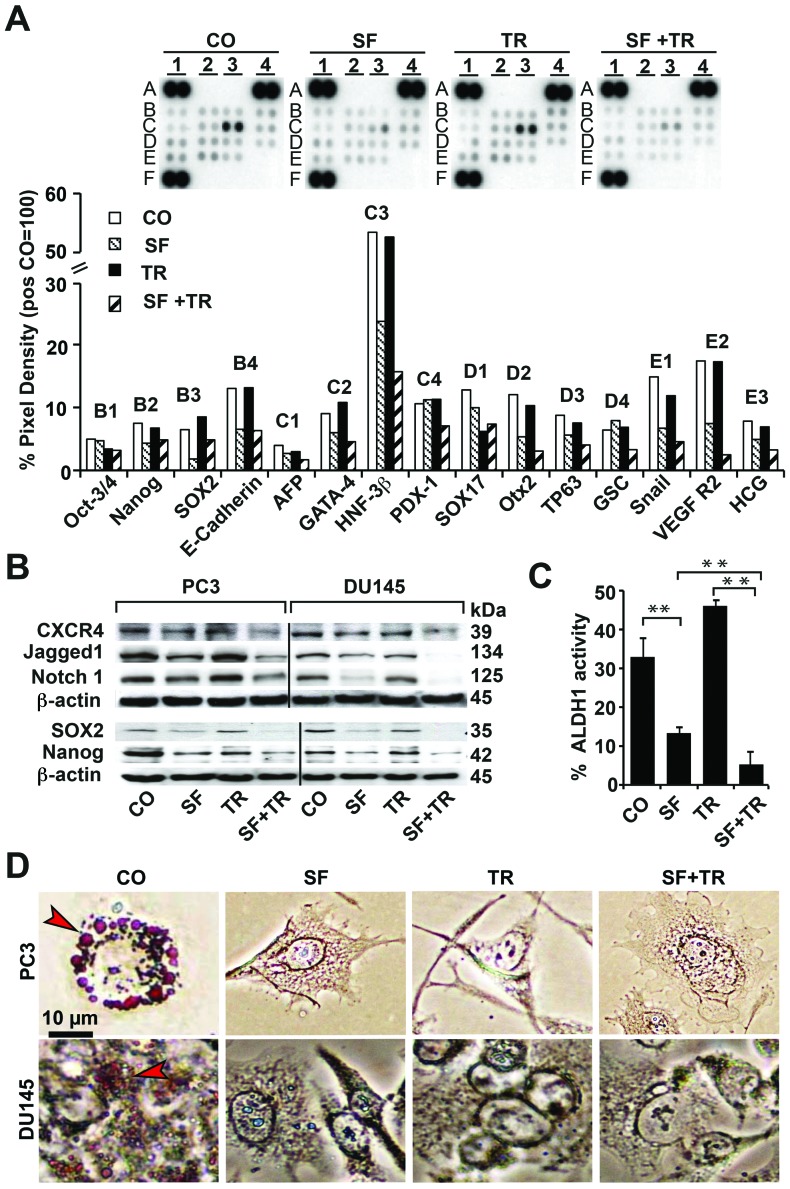
Sulforaphane, but not TRAIL, strongly inhibits stem cell-associated signaling and differentiation and their combination enhances these effects. (A) PC3 cells were treated as described in Fig. 1A. Twenty-four hours later, the proteins were isolated and a human pluripotent stem cell array was performed. The binding of proteins to antibodies spotted in duplicate on the membranes was detected using biotinylated secondary antibodies, streptavidin-HRP and chemiluminescence. After normalization to reference spots (positive control A1, A4 and F1; PBS-negative control E4), the pixel density was quantified using ImageJ software. (B) Proteins were also harvested, and a western blot analysis was performed to detect the expression of CXCR4, Jagged1, Notch 1, SOX2 and Nanog. The detection of β-actin served as a control for equal conditions. (C) For ALDH1 evaluation, PC3 cells were treated as described in Fig. 1A; the activity of ALDH1 was determined by a substrate assay and the turnover was analyzed by flow cytometry. The data are presented as the percentage of ALDH1-positive cells ± SD (^*^p<0.05, ^**^p<0.01). (D) DU145 and PC3 cells were treated as described in Fig. 1A. Twenty-four hours later, the medium was exchanged for NH AdipoDiff medium to induce adipocyte differentiation. After 14 days, the cells were stained with Oil Red O to detect the fat droplets in adipocytes. Representative images are shown, and the arrows indicate the red fat droplets. The scale bar indicates 10 *μ*m.

To study the influence of sulforaphane and TRAIL on stem cell signaling, we performed an antibody protein array to detect the levels of human pluripotent stem cell markers. PC3 cells were treated; the proteins were isolated after 24 h and incubated with the array membranes (Fig. 3A). TRAIL showed no or minor effects, whereas sulforaphane reduced the amount of Nanog, Sox2, E-cadherin, GATA-4, HNF-3β, SOX17, Otx2, TP63, Snail, VEGF R2 and HCG. Moreover, the combination treatment further reduced the levels of Oct-3/4, HNF-3β, PDX-1, Otx2, TP63, GSC, Snail, VEGF R2 and HCG. These results demonstrate that sulforaphane, particularly in combination with TRAIL, reduces the levels of proteins required for self-renewal, differentiation, cell migration, the epithelialmesenchymal transition (EMT) and tumorigenesis (26–41) (Table II). To further elucidate the obtained array results, we examined CSC marker proteins in the PC3 and DU145 cells by a western blot analysis (Fig. 3B). The expression of the CXCR4 receptor, which is involved in migration and metastasis (42), was inhibited following the sulforaphane-only treatment and the combination with TRAIL further reduced the expression. Similar results were found for the Notch 1 receptor and its ligand Jagged1, which are regulators of asymmetric and symmetric division, progression, and metastasis in prostate cancer (43). Correspondingly, sulforaphane, but not TRAIL, inhibited the expression of SOX2 and Nanog, which are important regulators of self-renewal potential (28), and the effects were stronger with the combination. Because ALDH1 activity has been reported to be required for self-renewal potential (44), we examined the enzymatic activity using a substrate assay and flow cytometry in PC3 cells (Fig. 3C). Sulforaphane significantly reduced the ALDH1 activity from ∼30 to 12%; conversely, TRAIL increased the activity to 45% and the combination treatment to 5%. We next evaluated the influence of the treatments on the differentiation potential of PC3 and DU145 cells; after treatment, the cell culture medium was exchanged for an adipocytic differentiation medium, and the formation of adipocytes was evaluated by staining the cells with Oil Red O after fourteen days (Fig. 3D). Although the untreated cells exhibited a high percentage of red cells, reflecting fat droplets, such fat droplets were completely absent in all the treatment groups, indicating that sulforaphane and TRAIL inhibited the cell differentiation potential. These data suggest that sulforaphane strongly inhibits stem cell signaling, ALDH1 activity, and differentiation potential, though TRAIL has only minor effects; both agents in combination resulted in the strongest effect.

### The combination of sulforaphane and TRAIL is superior to single treatments in reducing tumor growth and CSC marker expression in a xenograft model

**Figure 4.**
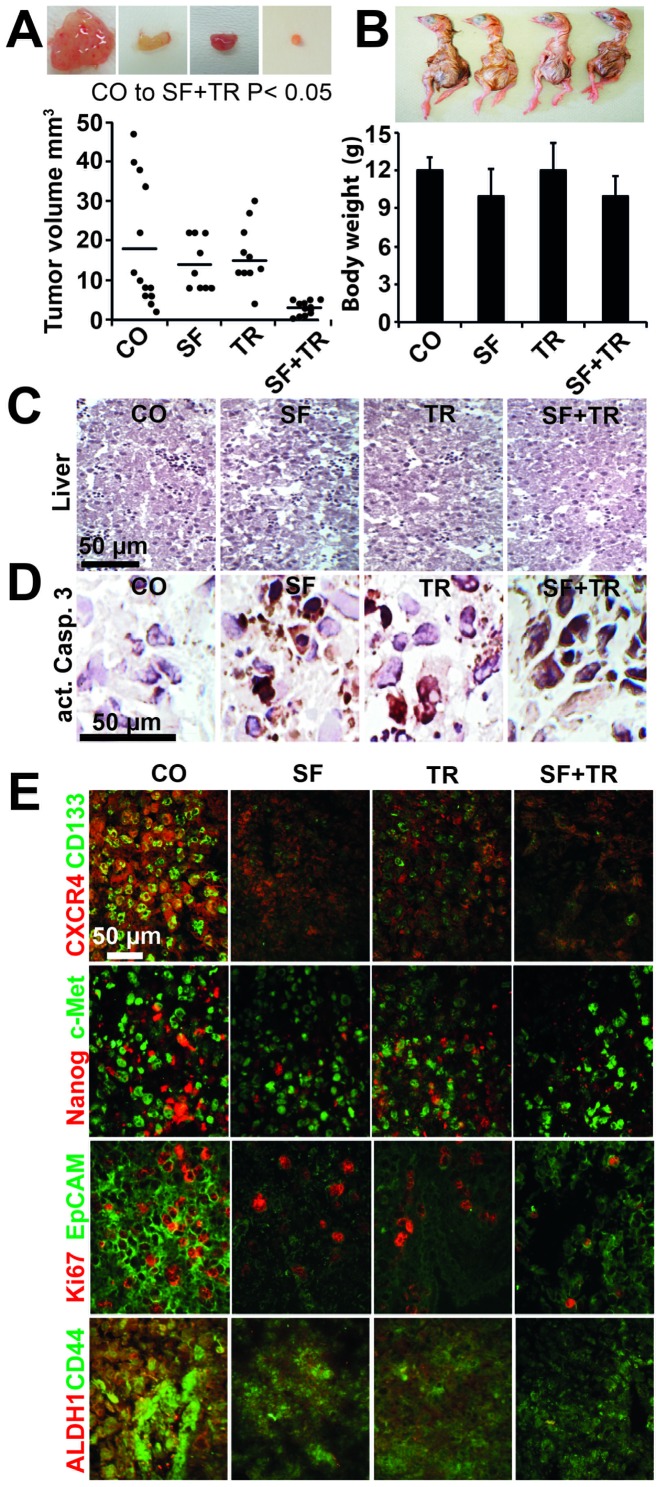
TRAIL and sulforaphane inhibit tumor growth *in vivo* and reduce CSC marker expression, with the strongest effects after their combination. (A) Untreated PC3 cells in Matrigel were transplanted into a plastic ring on the chorioallantoic membrane of fertilized chicken eggs at day 9 of embryonic development. At day 11, a 1-cm^2^ Whatman paper saturated with 10 *μ*M sulforaphane was placed directly adjacent to the tumor plastic ring. At day 12, a 5 *μ*g/ml TRAIL solution was dropped onto the Whatman paper until saturation. The tumor xenografts were resected at day 18 of embryonic development, and the volumes were determined as described in Materials and methods. The volumes of the individual tumors per group are presented as black dots and the bars indicate the average tumor size of each group. Representative images of the resected tumors are shown in the upper panel. (B) After tumor resection, the morphology and the body weights of the chicken embryos were evaluated. The average body weights of the embryos per group are presented in the diagram, and images of representative chicken embryos are shown. (C) The livers of the embryos were sectioned, and H&E staining was performed and visualized using microscopy. (D) Slices of the tumor tissue were immunohistochemically stained with an antibody for the detection of the cleaved fragment of active caspase-3 and the signal was detected using microscopy. (E) Slices of tumor tissue were double-immunofluorescence stained with antibodies for the detection of CD133/CXCR4, Nanog/c-Met, Ki67/EpCAM, and ALDH1/CD44 and positive signals were detected by fluorescence microscopy. The bar indicates 50 *μ*m.

To evaluate whether sulforaphane, TRAIL, and both agents together inhibit tumor growth and stem cell signaling in *in vivo*-treated xenografts, we transplanted untreated PC3 cells at day 9 of embryonic development into fertilized chicken eggs. The eggs were treated with sulforaphane at day 11, followed by treatment with TRAIL at day 12, and the xenograft tumors were resected at day 18. The average volume of the untreated xenograft tumors was 20 mm^3^, and sulforaphane or TRAIL reduced this to ∼15 mm^3^ (Fig. 4A). However, the combination of sulforaphane and TRAIL nearly abolished tumor growth, with the average volume reduced to 4 mm^3^. The analysis of the body weight of the chicken embryos revealed no significant difference between the treatment groups and no developmental defects were detectable (Fig. 4B). Similarly, liver necrosis did not occur, as evaluated by H&E staining of embryonal liver sections and microscopy (Fig. 4C). These results indicate that the treatment was well tolerated *in vivo*, without the induction of obvious side effects in the chicken embryos. Next, we analyzed apoptosis induction by staining the tumor sections with an antibody specific for the cleaved fragment of activated caspase-3 (Fig. 4D) and found that caspase-3 activity was increased in all the treatment groups but was strongest following the combined treatment. The tumor tissue was further analyzed by double immunofluorescence staining (Fig. 4E), which demonstrated that sulforaphane and TRAIL reduced the expression of the CSC markers CD133, CXCR4, Nanog, c-Met, EpCAM, CD44, and ALDH1 and the proliferation marker Ki67; the inhibition was much more pronounced with the combination of sulforaphane and TRAIL. These results indicate that sulforaphane and TRAIL reduce tumor growth *in vivo*, that their combination shows the strongest effects, without obvious side effects, and that the observed results are associated with the inhibition of CSC markers.

### The combination of sulforaphane and TRAIL is superior to single treatments in reducing the growth and stem cell marker expression of primary prostate CSCs

**Figure 5.**
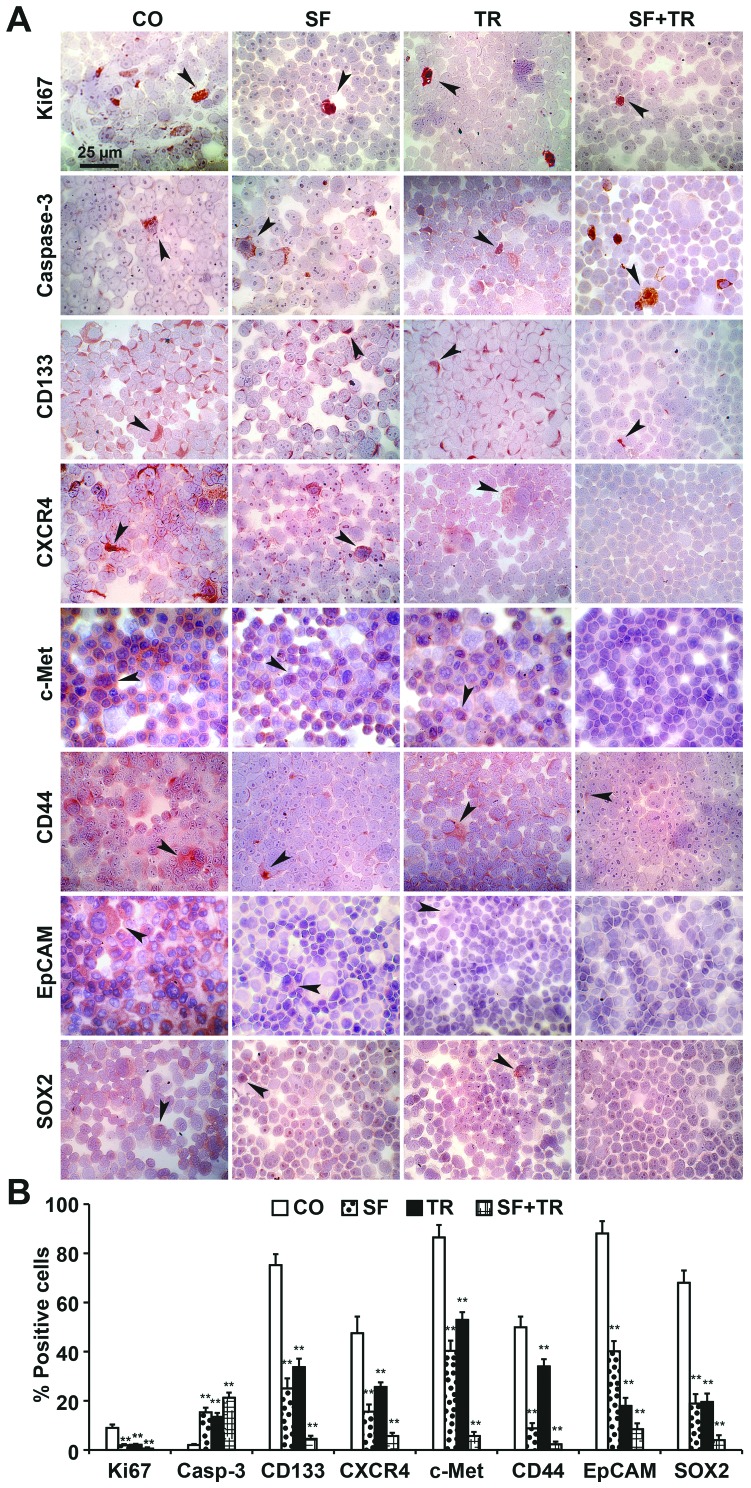
TRAIL and sulforaphane eliminate primary prostate CSCs synergistically via the induction of apoptosis and inhibition of proliferation and CSC marker expression. (A) Primary prostate CSCs were treated as described in Fig. 1A. Twenty-four hours after TRAIL treatment, the cells were cytospinned onto glass slides, and immunohistochemistry staining for proliferation (Ki67), apoptosis (cleaved fragment of active caspase-3), and CSC markers (CD133, CXCR4, c-Met, CD44, EpCAM and SOX2) was performed and visualized using microscopy. Representative images are shown and the arrows indicate positive cells. The bar indicates 25 *μ*m. (B) The number of positive cells after each staining procedure was quantified in 10 vision fields under ×400 magnification, and the means ± SD are shown. ^**^p<0.01.

To evaluate the effect of sulforaphane and TRAIL in a more patient-related model, we treated primary prostate CSCs that were directly derived from patient tumors. Following the treatment, the cells were cytospinned onto microscope slides, and positive cells with enhanced apoptosis or inhibited proliferation and CSC marker expression were detected by immunohistochemistry (Fig. 5A). The amount of positive cells was quantified by the evaluation of the number of positive-stained cells in 10 vision fields. Fig. 5B shows representative images and a diagram of the enhanced levels of the apoptosis marker ‘cleaved fragment of active caspase-3’ and reduced levels of the proliferation marker Ki67 and the CSC markers CD133, CXCR4, c-Met, CD44, EpCAM and SOX2. Each single treatment was effective, though the combination of sulforaphane and TRAIL exhibited the strongest effects. These data strengthen our concept that a nutritional strategy for enhanced sulforaphane intake could enhance the efficacy of TRAIL in attacking prostate CSCs.

## Discussion

TRAIL is selectively toxic to malignant, but not to non-malignant, cells and therefore is a promising anticancer agent (5). We examined whether TRAIL is able to eliminate AIPC cell lines enriched in CSC features and primary prostate CSCs. We found an induction of NF-κB activity by TRAIL that was associated with a minor efficacy of TRAIL in inhibiting clonogenicity, tumor engraftment and growth, spheroid formation and CSC signaling. In contrast, the treatment of cells with sulforaphane was largely more effective than TRAIL in eliminating CSC features. Furthermore, the combination of TRAIL with sulforaphane completely prevented basal TRAIL-induced NF-κB activity and synergistically led to a nearly complete elimination of CSCs. This result is in accordance with our recent data obtained for pancreatic CSCs, which exhibited enhanced basal NF-κB activity due to increased binding of the c-Rel and RelA subunits, which was further induced by TRAIL (9). Because the downregulation of c-Rel by siRNA restores sensitivity to TRAIL-induced apoptosis, the NF-κB prosurvival response induced by TRAIL may compete with its proapoptotic signals, and NF-κB activity may be a crucial regulator of the cellular outcome of survival or suicide.

Our present study supports the recent idea that prostate CSCs are resistant to TRAIL-induced apoptosis (6). This finding is in agreement with a prior study that reported that the TRAIL resistance of glioblastoma CSCs was due to the insufficient expression of the death receptors DR4 and DR5 and the inhibition of the CD95/Fas domain (45). In the same study, TRAIL resistance was circumvented by additional cisplatin treatment, which restored the expression of death receptors and Fas domain activity (45). Although our report does not address the expression of TRAIL receptors, we cite a paper that demonstrated the upregulation of the death receptors DR4 and DR5 in TRAIL-resistant PC3 and LNCap prostate cancer cells by sulforaphane, which enhanced TRAIL-induced apoptosis *in vitro* and in orthotopically growing PC3 xenografts transplanted into the prostate gland of immunodeficient mice (8).

For our *in vivo* studies, we used the xenotransplantation of PC3 cells into the CAM of fertilized chicken eggs. TRAIL was capable of reducing tumor engraftment and tumor growth to ∼50%, demonstrating the reduction of tumorigenic cells. Sulforaphane had similar effects *in vivo*, and tumor growth was completely inhibited by the combination treatment. These results suggest that the inhibition of TRAIL-induced survival signaling by sulforaphane might have switched the TRAIL response to apoptosis induction and that this, together with the cytotoxicity of sulforaphane, resulted in synergism and the complete elimination of the tumorigenic cells. Most importantly, neither the single treatments nor the combined agents had any obvious side effects on the chicken embryos, as developmental defects, weight loss or liver necrosis did not occur. In contrast, caspase-3 activity was induced, and proliferation and the expression of CSC markers CD133, CXCR4, c-Met, Nanog, EpCAM, CD44 and ALDH1 were almost completely abolished after the *in ovo* treatment of the PC3 xenograft tumors. These effects may be due to the observed interference of sulforaphane with NF-κB activity, an assumption that is underscored by the recent finding that tumor-initiating stem-like cells in human prostate cancer exhibit increased NF-κB signaling (46).

In our *in vitro* studies, we employed an antibody protein array and western blot analysis to confirmed the above described *in vivo* data. Our results showed that sulforaphane strongly inhibits the expression of the CSC proteins Nanog, SOX2, CXCR4, Jagged1, and Notch 1 and that of Snail, a mediator of the epithelial-mesenchymal transition (47). In addition, we found a sulforaphane-mediated inhibition of ALDH1 activity, which is known to be high in tumor-initiating and metastasis-initiating cells in human prostate cancer (16). Although TRAIL was not effective in reducing these progression markers, it synergistically enhanced the effect of sulforaphane in combination.

The targeting of prostate CSCs by sulforaphane may be expected to occur in patients, as exemplified by two recent epidemiological studies. Data from a prospective Canadian epidemiological study suggest that a high consumption of cruciferous vegetables, which contain high amounts of sulforaphane and related bioactive agents, is associated with the inhibition of metastasis (48). In the latter study, the dietary patterns of 1138 men with prostate cancer were evaluated, with a median follow-up of 4.2 years. Although the consumption of vegetables in general significantly reduced the relative risk (RR) of metastasis to 0.41, cruciferous vegetables showed the highest effect among all vegetables (crucifers in general RR=0.60; cabbage RR=0.64; cauliflower RR=0.48; broccoli RR= 0.55); of the crucifers, broccoli and cauliflower were most effective, with a significant effect at almost 1 serving per week and 3–5 servings being more effective. In a similar study, Richman and colleagues (49) prospectively examined the post-diagnostic intake of vegetables, particularly cruciferous vegetables, and the relationship with prostate cancer progression risk. These authors reported a 59% reduced risk of prostate cancer progression following cruciferous vegetable intake in men after diagnosis, with an inverse association for total vegetables that was not significant.

For future therapeutic application of our experimental strategy, we suggest a dietary intervention together with the targeted delivery of a protected TRAIL protein to tumors to avoid dilution in the body or inactivity due to the short half-life. In this regard, we are currently evaluating the suitability of the transduction of mesenchymal stem cells with TRAIL-expressing oncolytic adenoviruses. The supporting cytotoxicity of oncolytic adenoviruses may shift the balance of TRAIL signaling from survival to tumor-specific apoptosis.

## Figures and Tables

**Table I.** CSC characteristics of established human AIPC cell lines.

	DU145	PC3	Ref(s).
ATCC no.	HTB-81	CRL-1435	ATCC
Source	Brain metastasis	Bone metastasis, grade IV	ATCC
p53 status	Mutant	Absent	(14)
Growth characteristics	Adherent	Adherent	ATCC
Colony-forming capacity	High	High	(15)
Spheroid-formation capacity	None	None	(9)
ALDH activity	2.4±0.3	6.3±1.0	(16)
Growth in nude mice	Rapid, 100 CD44^+^/CD24^−^ cells form a tumor in 5 of 5 mice within 80 days	Rapid, 100 PC3 cells form a tumor in 1 of 8 mice within 90 days	(17,18)
CD44^+^/CD24^−^	7–10%	Detectable	(9,17,19,20)
CD133	0.01%	Setectable	(15,21)
α2β1	1–10%	100%	(22)
Invasiveness in Matrigel	> 20%, highly invasive	Highly invasive	(23,24)
E-cadherin expression	Low	Low	(23,25)

**Table II.** Human pluripotent stem cell antibody array.

Marker	Function	Ref.
AFP	α-fetoprotein, the fetal form of serum albumin, a tumor marker for hepatocellular carcinoma, germ cell tumors and metastatic liver cancer.	(26)
Oct-3/4	Transcription factor involved in self-renewal. Associated with an undifferentiated phenotype and tumors.	(27)
NANOG	Transcription factor involved in maintaining the pluripotency of stem cells.	(28)
SOX2	Transcription factor essential for the self-renewal and pluripotency of stem cells.	(29)
E-cadherin	Cell-cell adhesion glycoprotein. Loss of function contributes to the progression of cancer by increasing proliferation and invasion.	(30)
GATA-4	Zinc-finger transcription factor important in differentiation and embryogenesis. Decreased GATA-4 expression has been associated with carcinogenesis.	(31)
FOXA2	DNA-binding protein, the dysregulation of which has been linked to inflammation, tumorigenesis, and EMT.	(32, 33)
PDX-1	Transcription factor involved in the reprogramming of differentiated mouse pancreatic exocrine cells into β-cells.	(34)
SOX17	Transcription factor necessary for differentiation and the antagonization of self-renewal.	(35)
Otx2	Orthodenticle homeobox 2 is a homeobox transcription factor that controls brain morphogenesis and brain development.	(36)
p63	Member of the p53 family that is important for development and interactions between mesenchyme and epithelium. Loss may be involved in tumorigenesis.	(37)
GSC	The goosecoid homeobox gene is repressed during stem cell differentiation.	(38)
Snail	Zinc-finger transcription factor involved in inducing EMT during cancer progression via the downregulation of E-cadherin.	(39)
VEGF R2	Main receptor for VEGF involved in vasculogenesis, angiogenesis, proliferation, migration, survival and increased permeability.	(40)
hCG	Peptide hormone that meditates immune tolerance in pregnancy and tumors.	(41)

## Abbreviations:

AIPC: androgen-independent prostate cancer;
CSC: cancer stem cell
